# Physicians’ experiences and perceptions about withholding and withdrawal life-sustaining treatment in Chiang Mai University Hospital: a cross-sectional study

**DOI:** 10.1186/s12904-024-01511-6

**Published:** 2024-08-13

**Authors:** Nattanit Ketchaikosol, Kanokporn Pinyopornpanish, Chaisiri Angkurawaranon, Nisachol Dejkriengkraikul, Lalita Chutarattanakul

**Affiliations:** 1 Department of Family Medicine, Faculty of Medicine, Chiang Mai University, 110 Intawaroros Rd. Si Phum, Muang, Chiang Mai, Chiang Mai, 50200 Thailand; 2 Global Health and Chronic Conditions Research Group, Chiang Mai University, Chiang Mai, Thailand

**Keywords:** Physician experience, Decision making, Withholding life-sustaining treatment, Withdrawal life-sustaining treatment, End of life care, Palliative care, Practical experience

## Abstract

**Background:**

Withholding or withdrawing life-sustaining treatment in end-of-life patients is a challenging ethical issue faced by physicians. Understanding physicians’ experiences and factors influencing their decisions can lead to improvement in end-of-life care.

**Objectives:**

To investigate the experiences of Thai physicians when making decisions regarding the withholding or withdrawal of life-sustaining treatments in end-of-life situations. Additionally, the study aims to assess the consensus among physicians regarding the factors that influence these decisions and to explore the influence of families or surrogates on the decision-making process of physicians, utilizing case-based surveys.

**Methods:**

A web-based survey was conducted among physicians practicing in Chiang Mai University Hospital (June - October 2022).

**Results:**

Among 251 physicians (response rate 38.3%), most of the respondents (60.6%) reported that they experienced withholding or withdrawal treatment in end-of-life patients. Factors that influence their decision-making include patient’s preferences (100%), prognosis (93.4%), patients’ quality of life (92.8%), treatment burden (89.5%), and families’ request (87.5%). For a chronic disease with comatose condition, the majority of the physicians (47%) chose to continue treatments, including cardiopulmonary resuscitation (CPR). In contrast, only 2 physicians (0.8%) would do everything, in cases when families or surrogates insisted on stopping the treatment. This increased to 78.1% if the families insisted on continuing treatment.

**Conclusion:**

Withholding and withdrawal of life-sustaining treatments are common in Thailand. The key factors influencing their decision-making process included patient’s preferences and medical conditions and families’ requests. Effective communication and early engagement in advanced care planning between physicians, patients, and families empower them to align treatment choices with personal values.

**Supplementary Information:**

The online version contains supplementary material available at 10.1186/s12904-024-01511-6.

## Introduction

Over the past few decades, the care and support provided to terminally ill patients have been influenced by the incorporation of palliative care [1–4]. The principle of withholding and withdrawal life-sustaining treatment has been also increasingly integrated into end-of-life care. Life-sustaining treatment was traditionally administered to critically ill patients for the purpose of preserving life such as cardiopulmonary resuscitation (CPR), mechanical ventilator, hemodialysis, and antibiotics. Its utility has been challenged in cases of patients dealing with incurable diseases. Consequently, life-sustaining treatment is often considered futile in these circumstances. As a result, a decision to limit life-sustaining treatment can be made to alleviate the patient’s suffering and enhance the quality of life for both the patient and their family [5–7].

Decisions regarding the withholding or withdrawal of life-sustaining treatment require collaboration between the patient and their relatives. Physicians play a crucial role in communicating with patients about prognosis and treatment options [8]. When patients and relatives receive comprehensive information, decisions are made based on the patient’s preferences, leading to an improved quality of life and better outcomes [9, 10]. However, decision-making about treatment limitations remains controversial due to ethical dilemmas and legal issues [11].

While there are guidelines that help in decision-making, including laws that support the right to refuse medical treatment. Limiting life-sustaining treatment remains challenging for physicians. They must consider numerous factors, such as the patient’s autonomy in decision-making, medical standards, and their own beliefs [12, 13]. The personal experiences and perceptions of physicians influence the care they provide [14].

Previous studies have shown that physicians in Asian countries choose to withhold and withdraw life support treatment less than Western medicine [15]. Western countries generally adopt a more permissive and legally accepted approach to withholding and withdrawing life-sustaining treatment. For example, this is the case in the UK, Germany, the Netherlands, and Australia [16]. Legal frameworks and guidelines typically support this view, emphasizing patient autonomy and the importance of advance directives [17]. In contrast, Asian countries have more variability and a lower prevalence of these practices [18]. For instance, Japan and South Korea have complex approaches to withdrawing life-sustaining treatment, shaped by legal precedents and cultural factors [17]. In Japan, despite existing guidelines, there is still legal uncertainty and hesitation among healthcare providers due to past legal cases. In South Korea, landmark cases like Boramae Hospital and Severance Hospital have influenced guidelines but also highlighted the tension between legal requirements and medical practices. Consequently, these countries tend to have more cautious practices, influenced by legal, cultural, and social factors.

In Thailand, The National Health Act, B.E. 2007, allows individuals to create a living will to refuse life-prolonging treatments or alleviate severe suffering in terminal stages. It permits appointing a surrogate decision maker through an advance directive to make decisions for incapacitated patients. Physicians are vital in providing palliative care from diagnosis to end-of-life. The Act emphasizes the importance of informed decision-making by patients, relatives, or proxies about withholding or withdrawing life-sustaining treatment, clarifying that these actions are not considered euthanasia [19]. The primary objective of this study is to investigate the experiences of Thai physicians when making decisions regarding the withholding or withdrawal of life-sustaining treatments in end-of-life care. Additionally, the study aims to examine physicians’ consensus on the factors that influence decision-making. Finally, it seeks to explore the impact of family influence on physicians’ decision-making through a case-based survey.

## Methods

### Participants

This cross-sectional study was conducted among physicians who worked at Chiang Mai University Hospital during the period spanning June 2022 to October 2022. A web-based questionnaire was employed to collect the data. It was distributed to all physicians at Chiang Mai University Hospital, a tertiary care hospital in northern Thailand, through email addresses obtained from the hospital’s database. All participants provided informed consent before completing the questionnaire.

To estimate the sample size, applying the formula:$\:n=\frac{N}{(1+{Ne}^{2})\:}$ where n is the sample size, N is the total population size, and e is the margin of error (expressed as a proportion). The total number of physicians actively practicing at Chiang Mai University Hospital amounted to 655 individuals, and a margin of error (e) of 0.05. The calculated sample size was determined to be 248.

### Questionnaires

The questionnaire used in this study was developed based on a study published in January 2015^20^. It consisted of three parts as shown in Supplementary file 1. The first part of the questionnaire consisted of respondents’ demographic characteristics. In the second part, we assessed experiences with withholding and withdrawing life-sustaining treatments and physicians’ consensus on the factors influencing decision-making. These factors encompassed patient-related aspects, the care context, communication attitudes with patients and their families or surrogates, as well as perceptions of legal risk exposure. The evaluation employed a Likert scale, where the five response options are categorized into two categories. Specifically, ‘almost always’ and ‘often’ are combined into one category, while ‘sometimes,’ ‘seldom,’ and ‘almost never’ constitute another category. Similarly, ‘strongly agree’ and ‘agree’ are grouped together, while ‘neither agree nor disagree,’ ‘disagree,’ and ‘strongly disagree’ form a separate category.

The third section of the questionnaire aimed to investigate the influence of family members on physicians’ decision-making, utilizing a case-based survey. There were six potential choices related to the decision regarding withholding and/or withdrawing life-sustaining treatment, including full active treatment, including CPR (no withholding or withdrawal of treatment), continuing the most active treatment but excluding CPR (withholding CPR), continuing the current treatment but refraining from administering further complex treatments like hemodialysis or surgery (withholding treatment), continuing the current treatment but abstaining from additional treatments, such as antibiotics for sepsis (withholding treatment), discontinuing all treatments except mechanical ventilation (withdrawal of treatment), discontinuing the mechanical ventilator (withdrawal of treatment) and consulting the ethics committee. We utilized a case scenario translated into Thai version from a previous study [20] with permission from Prof. Dr. Younsuck Koh. Additionally, we conducted both forward and backward translations of the questionnaires. The back-translated version was sent to the original author, Prof. Dr. Younsuck Koh, for final approval.

### Data collection

An initial email containing an invitation message, informed consent form, and the link to the web-based questionnaire was sent to all eligible participants. For participants who did not respond within the two-week period, a follow-up email was sent. If the participant did not respond within one month after the second email, they were noted as non-respondents.

### Statistical analysis

Statistical analyses were performed using Stata statistical software version 16. Descriptive statistics were employed to summarize the data. Continuous variables were described using mean and standard deviation. Categorical variables were presented as frequency and percentage.

## Results

### Respondent characteristics

A total of 655 physicians were invited, with 251 completing the full survey, resulting in a response rate of 38.3%. Among all respondents, 51.4% were female. The respondents’ ages varied from 24 to 65 years, with an average age of 34.3 years (SD = 0.6). Their professional backgrounds were diverse including 31.4% in Internal Medicine, 23.1% in Surgery, 12.3% in Family Medicine, and 27.2% in other specialties. The characteristics of the respondents are described in Table 1.

**Table 1:** Demographic data of respondents

Characteristics	*N* = 251
Age, mean ± SD	34.3 ± 0.6
Female gender, n (%)	129 (51.4)
Specialty, n (%)	
Internal Medicine Surgery Family Medicine Otolaryngology Obstetrics and Gynecology Anesthesiology Others	79 (31.4) 58 (23.1) 31 (12.3) 24 (9.6) 19 (7.6) 14 (5.6) 24 (10.4)
Designation of occupation, n (%)	
Intern Resident Fellowship Staff specialist	79 (31.5) 61 (24.3) 3 (1.2) 108 (43.0)
Numbers of years of practice, mean ± SD	6.9 ± 0.6
Experience in almost always/often withholding or withdrawing treatment for patients with no real chance of recovering a meaningful life, n (%)	
- Withholding or withdrawing - Withholding only - Withdrawing only - Neither withholding nor withdrawing	152 (60.6) 145 (57.8) 47 (18.7) 99 (39.4)
Perception ethical difference between withholding and withdrawing treatments, n (%)	136 (54.2)

### Experiences and physicians’ consensus on the factors that influence decision-making in end-of-life care

Most physicians (60.6%) reported almost always or often withholding or withdrawing treatment for patients with no real chance of recovering a meaningful life. Nonetheless, most of them (54.2%) felt that there was an ethical difference between withholding and withdrawal treatment.

As shown in Table 2, among physicians who have experienced withholding or withdrawing treatment, almost all physicians reported agreement that the patient’s preferences and families’ requests were important factors in withholding or withdrawing treatment (100% and 87.5%, respectively). Despite this, only 48% of physicians felt comfortable talking to the family about limiting life-sustaining treatment. Furthermore, legal risk exposure was another factor taken into consideration in end-of-life care decision-making (26.3%).

**Table 2:** Physicians’ consensus on the factors that influence decision-making in end-of-life care

Factors	*N*, (%)
**Patient-related factors**	**Strongly agree or agree**
The patient’s preferences.	152 (100)
The disease has no chance of being cured or no long-term survival.	142 (93.4)
The expected long-term quality of life.	141 (92.8)
Patients are expected to suffer if treatment continues in the ICU.	136 (89.5)
**Context of care**	**Strongly agree or agree**
Families’ or surrogate’s requests.	133 (87.5)
The patient has financial problems.	49 (32.2)
The ICU bed is almost reached limitation.	14 (9.2)
Financial impact on the hospital.	13 (8.6)
**Perception of communication with patients and families/surrogates**	**Almost always or often**
Physicians are comfortable talking to the family about withholding or withdrawing life-sustaining treatment.	73 (48.0)
Patient or surrogate request inappropriate life-sustaining treatment.	43 (28.3)
**Perception of exposure to legal risk with the practice**	**Almost always or often**
Withholding or withdrawing treatment	40 (26.3)

### The influence of families or surrogates on end-of-life care practices in different situations

Table 3 describes the management in different situations based on providing care for a 50-year-old male with a chronic disease in a comatose state after cardiac arrest. In the absence of families, surrogates, or advanced directives, the majority of the physicians (47%) chose to continue treatments, including cardiopulmonary resuscitation (CPR) in the event of a cardiac arrest. In contrast, only 2 physicians (0.8%) would do everything, in cases where families or surrogates insisted on stopping the treatment. This increased to 78.1% if the families insisted on continuing treatment. The results of decisions categorized by experience of withholding or withdrawing, job position, or years of practice are shown in Supplementary file 2.

**Table 3:** Withholding and withdrawing decisions based on the vignette in three situations

A 50-year-old male patient suffering from chronic obstructive pulmonary disease (COPD) for many years has been admitted repeatedly due to respiratory failure and has required repeated prolonged ventilatory support. This time he was suffering from respiratory failure again, along with prolonged cardiac arrest. After 72 h, he was still deeply comatose and required ventilatory support.	The patient did not have a family or an advanced directive.	The patient’s family insisted on stopping further treatment and withdrawing it.	The patient’s family insisted on continuing the most active treatment.
- Continue full active treatment, including CPR, if the patient has a cardiac arrest again.	118 (47.0%)	2 (0.8%)	196 (78.1%)
- Continue the most active treatment, but do not include CPR.	25 (10.0%)	10 (4.0%)	15 (6.0%)
- Continue the current treatment, but do not give further complicated treatments such as hemodialysis or surgery.	46 (18.3%)	63 (25.1%)	20 (7.9%)
- Continue the current treatment, but do not give further additional treatments, such as antibiotics, to treat sepsis.	31 (12.4%)	54 (21.5%)	9 (3.6%)
- Discontinue all treatments (intravenous fluid, nasogastric tube), except mechanical ventilation.	3 (1.2%)	58 (23.1%)	2 (0.8%)
- Discontinue the mechanical ventilator (allow the patient to die).	0 (0.0%)	52 (20.7%)	0 (0.0%)
- Consult the ethics committee.	28 (11.1%)	12 (4.8%)	9 (3.6%)

## Discussion

In a sample of physicians from various specialties working in a tertiary hospital in Thailand, we found that most of them have encountered situations involving the withholding and withdrawal of life-sustaining treatment. However, their experiences tend to involve withholding treatment more frequently than withdrawing it. Factors that physicians consider essential in determining the limitations of treatment include patient’s preferences and their medical condition. However, most physicians tend to rely on the families’ requests.

Even though palliative care has developed significantly in Thailand [21, 22], similarly in many countries, doctors still tend to choose to withhold more often than withdraw [20, 23]. This might be because doctors perceive that withdrawing is more psychologically difficult [24, 25] and ethically problematic. Furthermore, they believe that there are different ethical considerations between withholding and withdrawing treatment [24, 26]. This is consistent with the evidence in this study that most doctors believed there were different ethics between these two alternative treatments. In addition to the reasons mentioned above, religion [27, 28], law [29], and experience in caring for terminally ill patients also affect the decision-making process of either withholding or withdrawing treatment [30, 31]. The evidence also suggests that specialized education in palliative medicine could play a significant role for physicians in making less aggressive decisions regarding end-of-life care [32, 33].

This study found that patient’s preferences are the most important factor in decision-making which is consistent with various guidelines that focus on patient autonomy [34]. The second important factor is the patient’s condition. If the patient’s condition reaches a point where life-sustaining treatment becomes ineffective or unbeneficial, those treatments may be considered medically futile. Life-sustaining treatments may only prolong the dying process without providing any meaningful benefit [35]. Therefore, it is important for physicians to initiate early communication with patients, particularly those affected by incurable diseases. Physicians should involve patients in discussions about advance care plans, providing them to make well-informed decisions regarding their preferences for the end-of-life period. Furthermore, the ability to evaluate patients at the end of life is essential not only for effective communication but also for providing essential psychological and emotional support. Therefore, this approach ensures that the patient’s autonomy is followed.

Another aspect that respondents considered when making their decisions is families’ requests. This aligns with the practices in many Asian and Western countries where families play an essential role in the decision-making process at the end-of-life stage [20, 36]. However, a previous study shows that the family’s role in end-of-life decision-making in Asian countries (Japan, Korea, and Taiwan), particularly when compared with England, traditionally places a greater emphasis on families and communities rather than on individuals [17]. Families or surrogates often understand the patient’s beliefs and preferences. As a result, they may function as representatives of the patient, ensuring that the patient’s desires are respected when they are unable to make decisions.

The significance of family was also apparent in this study, as an example of a case scenario involving an incurable disease. Most physicians are likely to follow the families’ request if they insisted on discontinuing the treatment. On the other hand, few physicians will continue full life-sustaining treatment. This may be due to the lack of clear guidelines in Thailand on the limitations of treatment or the absence of advanced directives. Moreover, physicians may consider law or ethics issues that make decision-making more complicated [24, 26, 29]. Compared to another Asian survey [20], our respondents were also likely to rely on families’ or surrogates’ requests. In Hong Kong, where guidelines on life-sustaining treatment for the terminally ill are well established [37], there is a tendency for physicians to engage families more frequently in conversations concerning end-of-life matters compared to their European counterparts [38]. In comparison to the American Medical Association (AMA) guidelines [39] of the US on withholding or withdrawing life-sustaining treatment, both Hong Kong and US guidelines emphasize the importance of involving family and surrogate decision-makers in end-of-life decisions while prioritizing the patient’s wishes and decision-making capacity. However, the AMA guidelines emphasize the individual’s right to make decisions about their own health and well-being, strongly stating that physicians should explain that surrogates should make decisions to withhold or withdraw life-sustaining interventions only when the patient lacks decision-making capacity. While Hong Kong’s guidelines allow for more involvement of the family or surrogate in communication and aim to resolve disagreements between the patient’s and family’s decisions, they stipulate that the patient’s decision should not be overridden. In Thailand, the absence of clear guidelines for the inclusion of family members in the decision-making process regarding the withholding and withdrawing of life-sustaining treatment highlights the need for government or relevant authorities to establish and disseminate such guidelines to inform and support clinical practice. Additionally, hospital-level ethics committees may be useful in assisting with decision-making in complex situations.

Patients’ financial problems, the lack of ICU beds, and the financial impact on hospitals, although reported at lower rates, are critical factors in end-of-life care decision-making. These elements introduce significant ethical and practical challenges. Financial concerns among patients can significantly impact how physicians adjust their clinical decisions. It may lead to difficult choices about the intensity of treatments and the use of advance directives [40, 41]. For instance, in settings with limited resources, the high cost of prolonged life-sustaining treatments may give rise to ethical issues that influence the continuation or withdrawal of care. In addition, decisions influenced by the financial impact on the hospital can be controversial, as they may conflict with the primary objective of patient-centered care. This highlights the need for policies ensuring that all patients receive proper care regardless of their financial condition. It raises questions about equity and access to healthcare, as well as the importance of having robust guidelines and ethical frameworks to support physicians in making these challenging decisions.

Furthermore, it is interesting that while 57% of physicians have experienced withholding or withdrawing treatment, only 48% feel comfortable discussing it with families. This discrepancy suggests that while physicians may frequently encounter these situations, they often struggle with the communication aspect. This discomfort can be attributed to several factors, including fear of causing distress to families and personal ethical dilemmas [42]. Therefore, improving education about palliative care for doctors is crucial [43]. It is necessary to provide knowledge about communication skills, ethical decision-making, and importantly, understanding the concepts of withholding and withdrawing treatments. This can assist physicians in navigating these challenging conversations more comfortably.

In situations where the patient lacks decision-making capacity and has no available relatives or advance directive. Physicians may consult with ethics committees to ensure they are following regulations and ethical guidelines. These consultations can provide guidance on decision-making processes. Therefore, the development of guidelines is important to assist physicians in handling difficult situations.

There are several limitations to this study. Firstly, it was conducted in a single setting in Thailand, so the findings may not be applicable to other settings. Secondly, the study design is cross-sectional, which can introduce recall bias when respondents are asked about past events. However, this bias is expected to be rare and may only affect decision-making discomfort. Respondents who have experienced the events should be able to recall them accurately. Thirdly, there might be unrecorded details or factors beyond the reported information when considering different scenarios. Conducting more comprehensive and in-depth studies on decision-making issues would be beneficial.

On the other hand, the strength of this study lies in addressing an important and sensitive topic in medical practice, specifically the lack of clear guidelines in Thailand for making decisions about limiting life-sustaining treatments. The information gathered from this study can be used to develop plans and assist physicians in making informed decisions regarding the withdrawal and withholding of life-sustaining treatments in the future.

## Conclusion

Most physicians have experienced the withholding and withdrawal of life-sustaining treatment. From a Western bioethical perspective, there is no ethical difference between withholding and withdrawing life-sustaining treatments. However, physicians usually perceive these actions differently. Withdrawing treatment feels more like taking action, which can be harder emotionally, leading to a distinction in practice [16]. When considering the limitations of life-sustaining treatments in patients who were incapacitated and unable to make decisions, the first factor considered in the treatment choice was the patients’ preferences, followed by the requests of their relatives. There are some challenging situations, especially when resources are limited, and financial concerns arise. To support physicians in making these important decisions, there is a need for clear clinical practice guidelines and ethics, enhanced professional education, and improved communication skills.

### Electronic supplementary material

Below is the link to the electronic supplementary material.

Supplementary Material 1

Supplementary Material 2

## Data Availability

The datasets used and/or analyzed during the current study are available from the corresponding author (Lalita Chutarattanakul, MD.) on reasonable request.
