# Supplementary material for: Physicians’ experiences and perceptions about withholding and withdrawal life-sustaining treatment in Chiang Mai University Hospital: a cross-sectional study
Source: BMC Palliat Care. 2024 Aug 13;23:206. doi: 10.1186/s12904-024-01511-6 (PMC11320918; doi:10.1186/s12904-024-01511-6)
Supplement: Supplementary file 1 — Supplementary Material 1 [file 12904_2024_1511_MOESM1_ESM.docx]

**Questionnaires**

**Part 1: Background information**

1. Sex ⬜ Female ⬜ Male

2. Age ________ years old

3. Designation of occupation ⬜ Intern ⬜ Resident ⬜ Fellow

⬜ Staff specialist ⬜ Other (please specify) _______________

4. Primary specialty

⬜ Internal medicine ⬜ Surgery ⬜ Obstetrics/Gynecology ⬜ Anaesthesiology ⬜ Ophthalmology ⬜ Otolaryngology ⬜ Orthopedics ⬜ Emergency medicine ⬜ Family Medicine ⬜ Others (please specify) ___________

5. How long have you been working in your specialty? ________ years

6. Did you have experiences caring for terminally ill patients?

⬜ Yes ⬜ No

**Part 2:** **Withholding therapy and withdrawing therapy**

**Please acknowledge the definition described below:**

**Withholding therapy** is a decision not to start or increase a treatment e.g., do not start CPR, hemodialysis.

**Withdrawing therapy** is a decision to stop a treatment presently being given, e.g., termination of mechanical ventilation or intravenous fluid therapy.

**Life-Sustaining Treatment** is a medical treatment that supports or substitutes the function of vital organs such as cardiopulmonary resuscitation; CPR, intubation, vasopressors, hemodialysis, blood transfusion, and antibiotics.

7. How often do you experience withholding treatment for patients with no real chance of recovering a meaningful life?

⬜ Almost always ⬜ Often ⬜ Sometimes ⬜ Seldom ⬜ Almost never

8. How often do you experience withdrawing treatment for patients with no real chance of recovering a meaningful life?

⬜ Almost always ⬜ Often ⬜ Sometimes ⬜ Seldom ⬜ Almost never

9. Do you perceive ethical difference between withholding and withdrawing treatments?

⬜ Yes ⬜ No

10.) Do you feel comfortable talking to the patient's family about withholding and withdrawing life-sustaining treatment?

⬜ Almost always ⬜ Often ⬜ Sometimes ⬜ Seldom ⬜ Almost never

11. In your opinion, which following factors affect the consideration of requests for withholding or withdrawing life-sustaining treatment in critically ill/ end-of-life care patients?

|  | **Strongly agree** | **Agree** | **Neither agree nor disagree** | **Disagree** | **Strongly disagree** |
| --- | --- | --- | --- | --- | --- |
| **Patient-related factors** | | | | | |
| The patient’s preferences. |  |  |  |  |  |
| The disease has no chance of being cured or no long-term survival. |  |  |  |  |  |
| The expected long-term quality of life. |  |  |  |  |  |
| Patients are expected to suffer if treatment continues in the ICU. |  |  |  |  |  |
| **Context of care** | | | | | |
| Families’ or surrogate’s requests. |  |  |  |  |  |
| The patient has financial problems. |  |  |  |  |  |
| The ICU bed is almost reached limitation. |  |  |  |  |  |
| Financial impact on the hospital. |  |  |  |  |  |
| **Perception of communication with patients and families/surrogates** | | | | | |
| Physicians are comfortable talking to the family about withholding or withdrawing life-sustaining treatment. |  |  |  |  |  |
| Patient or surrogate request inappropriate life-sustaining treatment. |  |  |  |  |  |
| **Perception of exposure to legal risk with the practice** | | | | | |
| Withholding or withdrawing treatment |  |  |  |  |  |

**Part 3: Case scenario**

A 50-year-old male patient suffering from chronic obstructive pulmonary disease (COPD) for many years has been admitted repeatedly due to respiratory failure and has required repeated prolonged ventilatory support. This time he was suffering from respiratory failure again, along with prolonged cardiac arrest. After 72 hours, he was still deeply comatose and required ventilatory support. *Choose one answer for each of the following situations.*

**1) The patient does not have a family or advanced directive/living wills.**

□ Continue full active treatment, including Cardiopulmonary resuscitation (CPR) if the patient has a cardiac arrest again.

□ Continue the most active treatment but not include cardiopulmonary resuscitation (CPR).

□ Continue the current treatment, but do not give further complicated treatments such as hemodialysis or surgery.

□ Continue the current treatment, but do not give further additional treatments, such as antibiotics, to treat sepsis.

□ Discontinue all treatments (intravenous fluid, nasogastric tube), except mechanical ventilator.

□ Discontinue the mechanical ventilator (allow the patient to die).

□ Consult the ethics committee.

**2) The patient's family insists on stopping further treatment and withdrawing it.**

□ Continue full active treatment, including Cardiopulmonary resuscitation (CPR) if the patient has a cardiac arrest again.

□ Continue the most active treatment but not include cardiopulmonary resuscitation (CPR).

□ Continue the current treatment, but do not give further complicated treatments such as hemodialysis or surgery.

□ Continue the current treatment, but do not give further additional treatments, such as antibiotics, to treat sepsis.

□ Discontinue all treatments (intravenous fluid, nasogastric tube), except mechanical ventilator.

□ Discontinue the mechanical ventilator (allow the patient to die).

□ Consult the ethics committee.

**3) The patient's family insists on continuing the most active treatment.**

□ Continue full active treatment, including Cardiopulmonary resuscitation (CPR) if the patient has a cardiac arrest again.

□ Continue the most active treatment but not include cardiopulmonary resuscitation (CPR).

□ Continue the current treatment, but do not give further complicated treatments such as hemodialysis or surgery.

□ Continue the current treatment, but do not give further additional treatments, such as antibiotics, to treat sepsis.

□ Discontinue all treatments (intravenous fluid, nasogastric tube), except mechanical ventilator.

□ Discontinue the mechanical ventilator (allow the patient to die).

□ Consult the ethics committee.
