# Supplementary material for: Physicians’ experiences and perceptions about withholding and withdrawal life-sustaining treatment in Chiang Mai University Hospital: a cross-sectional study
Source: BMC Palliat Care. 2024 Aug 13;23:206. doi: 10.1186/s12904-024-01511-6 (PMC11320918; doi:10.1186/s12904-024-01511-6)
Supplement: Supplementary file 2 — Supplementary Material 2 [file 12904_2024_1511_MOESM2_ESM.docx]

**Supplementary table 1** Withholding and withdrawing decisions based on the vignette in three situations categorized by experience of withholding or withdrawing (Frequently VS never)

| A 50-year-old male patient suffering from chronic obstructive pulmonary disease (COPD) for many years has been admitted repeatedly due to respiratory failure and has required repeated prolonged ventilatory support. This time he was suffering from respiratory failure again, along with prolonged cardiac arrest. After 72 hours, he was still deeply comatose and required ventilatory support. | The patient did not have a family or an advanced directive. | | The patient's family insisted on stopping further treatment and withdrawing it. | | The patient's family insisted on continuing the most active treatment. | |
| --- | --- | --- | --- | --- | --- | --- |
| **Experience about WH/WD** | Frequently | Never | Frequently | Never | Frequently | Never |
| - Continue full active treatment, including CPR, if the patient has a cardiac arrest again. | 63  (41.4%) | 55  (55.5%) | 1  (0.6%) | 1  (1.0%) | 109 (71.7%) | 87  (87.8%) |
| - Continue the most active treatment, but do not include CPR. | 17  (11.1%) | 8  (8.0%) | 7  (4.6%) | 3  (3.0%) | 11  (7.2%) | 4  (4.0%) |
| - Continue the current treatment, but do not give further complicated treatments such as hemodialysis or surgery. | 35  (23.0%) | 11  (11.1%) | 36  (23.6%) | 27  (27.2%) | 17  (11.1%) | 3  (3.0%) |
| - Continue the current treatment, but do not give further additional treatments, such as antibiotics, to treat sepsis. | 22  (14.4%) | 9  (9.0%) | 32  (21.0%) | 22  (22.2%) | 7  (4.6%) | 2  (2.0%) |
| - Discontinue all treatments (intravenous fluid, nasogastric tube), except mechanical ventilation. | 3  (1.9%) | 0  (0.00%) | 38  (25.0%) | 20  (20.2%) | 1  (0.6%) | 1  (1.0%) |
| - Discontinue the mechanical ventilator (allow the patient to die). | 0  (0.00%) | 0  (0.00%) | 33  (21.7%) | 19  (19.1%) | 0  (0.00%) | 0  (0.00%) |
| - Consult the ethics committee. | 12  (7.8%) | 16  (16.1%) | 5  (3.2%) | 7  (7.0%) | 7  (4.6%) | 2  (2.0%) |

**Supplementary table 2** Withholding and withdrawing decisions based on the vignette in three situations categorized by job position (Staff VS trainees)

| A 50-year-old male patient suffering from chronic obstructive pulmonary disease (COPD) for many years has been admitted repeatedly due to respiratory failure and has required repeated prolonged ventilatory support. This time he was suffering from respiratory failure again, along with prolonged cardiac arrest. After 72 hours, he was still deeply comatose and required ventilatory support. | The patient did not have a family or an advanced directive. | | The patient's family insisted on stopping further treatment and withdrawing it. | | The patient's family insisted on continuing the most active treatment. | |
| --- | --- | --- | --- | --- | --- | --- |
| **Group of people** | Staff | Trainees | Staff | Trainees | Staff | Trainees |
| - Continue full active treatment, including CPR, if the patient has a cardiac arrest again. | 58  (53.7%) | 60  (41.9%) | 0  (0.00%) | 2  (1.4%) | 72  (66.6%) | 124  (86.7%) |
| - Continue the most active treatment, but do not include CPR. | 9  (8.3%) | 16  (11.1%) | 6  (5.5%) | 4  (2.8%) | 10  (9.2%) | 5  (3.5%) |
| - Continue the current treatment, but do not give further complicated treatments such as hemodialysis or surgery. | 17  (15.7%) | 29  (20.2%) | 27  (25.0%) | 36  (25.1%) | 12  (11.1%) | 8  (5.5%) |
| - Continue the current treatment, but do not give further additional treatments, such as antibiotics, to treat sepsis. | 12  (11.1%) | 19  (13.2%) | 29  (26.8%) | 25  (17.4%) | 6  (5.5%) | 3  (2.1%) |
| - Discontinue all treatments (intravenous fluid, nasogastric tube), except mechanical ventilation. | 2  (1.8%) | 1  (0.7%) | 26  (24.0%) | 32  (22.3%) | 0  (0.00%) | 2  (1.4%) |
| - Discontinue the mechanical ventilator (allow the patient to die). | 0  (0.00%) | 0  (0.00%) | 16  (14.8%) | 36  (25.1%) | 0  (0.00%) | 0  (0.00%) |
| - Consult the ethics committee. | 10  (9.2%) | 18  (12.5%) | 4  (3.7%) | 8  (5.5%) | 8  (7.4%) | 1  (0.7%) |

**Supplementary table 3** Withholding and withdrawing decisions based on the vignette in three situations categorized by years of practice (< 5 years VS > 5 years)

| A 50-year-old male patient suffering from chronic obstructive pulmonary disease (COPD) for many years has been admitted repeatedly due to respiratory failure and has required repeated prolonged ventilatory support. This time he was suffering from respiratory failure again, along with prolonged cardiac arrest. After 72 hours, he was still deeply comatose and required ventilatory support. | The patient did not have a family or an advanced directive. | | The patient's family insisted on stopping further treatment and withdrawing it. | | The patient's family insisted on continuing the most active treatment. | |
| --- | --- | --- | --- | --- | --- | --- |
| **Years of experience** | < 5 yr | > 5 yr | < 5 yr | > 5 yr | < 5 yr | > 5 yr |
| - Continue full active treatment, including CPR, if the patient has a cardiac arrest again. | 78  (46.4%) | 40  (48.1%) | 2  (1.1%) | 0  (0.00%) | 144  (85.1%) | 52  (62.6%) |
| - Continue the most active treatment, but do not include CPR. | 19  (11.3%) | 6  (7.2%) | 4  (2.3%) | 6  (7.2%) | 8  (4.7%) | 7  (8.4%) |
| - Continue the current treatment, but do not give further complicated treatments such as hemodialysis or surgery. | 29  (17.2%) | 17  (20.4%) | 47  (27.9%) | 16  (19.2%) | 9  (5.3%) | 11  (13.2%) |
| - Continue the current treatment, but do not give further additional treatments, such as antibiotics, to treat sepsis. | 20  (11.9%) | 11  (13.2%) | 29  (17.2%) | 25  (30.1%) | 3  (1.7%) | 6  (7.2%) |
| - Discontinue all treatments (intravenous fluid, nasogastric tube), except mechanical ventilation. | 2  (1.19%) | 1  (1.2%) | 40  (23.8%) | 18  (21.6%) | 2  (1.1%) | 0  (0.00%) |
| - Discontinue the mechanical ventilator (allow the patient to die). | 0  (0.00%) | 0  (0.00%) | 36  (21.4%) | 16  (19.2%) | 0  (0.00%) | 0  (0.00%) |
| - Consult the ethics committee. | 20  (11.9%) | 8  (9.6%) | 10  (5.9%) | 2  (2.4%) | 2  (1.1%) | 7  (8.4%) |
